# The Efficacy of Chinese Herbal Medicine as an Adjunctive Therapy for Advanced Non-small Cell Lung Cancer: A Systematic Review and Meta-analysis

**DOI:** 10.1371/journal.pone.0057604

**Published:** 2013-02-28

**Authors:** Shi Guang Li, Hai Yong Chen, Chen Sheng Ou-Yang, Xi-Xin Wang, Zhen-Jiang Yang, Yao Tong, William C.S. Cho

**Affiliations:** 1 Graduate School, Guangzhou University of Chinese Medicine, Guangzhou, China; 2 Department of Oncology and Hematology, Shenzhen Hospital of Traditional Chinese Medicine, Shenzhen, Guangdong Province, China; 3 School of Chinese Medicine, Li Ka Shing Faculty of Medicine, The University of Hong Kong, Hong Kong SAR, China; 4 Shanxi Province Hospital of Traditional Chinese Medicine, Taiyuan, Shanxi Province, China; 5 Department of Clinical Oncology, Queen Elizabeth Hospital, Hong Kong SAR, China; Univesity of Texas Southwestern Medical Center at Dallas, United States of America

## Abstract

Many published studies reflect the growing application of complementary and alternative medicine, particularly Chinese herbal medicine (CHM) use in combination with conventional cancer therapy for advanced non-small cell lung cancer (NSCLC), but its efficacy remains largely unexplored. The purpose of this study is to evaluate the efficacy of CHM combined with conventional chemotherapy (CT) in the treatment of advanced NSCLC. Publications in 11 electronic databases were extensively searched, and 24 trials were included for analysis. A sum of 2,109 patients was enrolled in these studies, at which 1,064 patients participated in CT combined CHM and 1,039 in CT (six patients dropped out and were not reported the group enrolled). Compared to using CT alone, CHM combined with CT significantly increase one-year survival rate (RR = 1.36, 95% CI = 1.15–1.60, p = 0.0003). Besides, the combined therapy significantly increased immediate tumor response (RR = 1.36, 95% CI = 1.19–1.56, p<1.0E−5) and improved Karnofsky performance score (KPS) (RR = 2.90, 95% CI = 1.62–5.18, p = 0.0003). Combined therapy remarkably reduced the nausea and vomiting at toxicity grade of III–IV (RR = 0.24, 95% CI = 0.12–0.50, p = 0.0001) and prevented the decline of hemoglobin and platelet in patients under CT at toxicity grade of I–IV (RR = 0.64, 95% CI = 0.51–0.80, p<0.0001). Moreover, the herbs that are frequently used in NSCLC patients were identified. This systematic review suggests that CHM as an adjuvant therapy can reduce CT toxicity, prolong survival rate, enhance immediate tumor response, and improve KPS in advanced NSCLC patients. However, due to the lack of large-scale randomized clinical trials in the included studies, further larger scale trials are needed.

## Introduction

Lung cancer is the most common malignancy worldwide and a leading cause of cancer-related deaths. In 2012, it is estimated that 160,300 deaths (87,700 in men, 72,600 in women) from lung cancer would occur in the United States [1].

The non-small cell lung cancer (NSCLC) is the most common form of lung cancer, which accounts for approximately 85% of all lung cancer cases. Nowadays the standard treatment for patient with advanced NSCLC who has a good performance status, platinum-based chemotherapy (CT) is the first-line regimen [2], [3]. However, platinum-based CT has the potential for severe adverse events, and the International Adjuvant Lung Cancer Trial suggests that more deaths in the CT group and the benefit of CT decreased over time [4], [5]. In spite of the development of new CT regimens use in the treatment of NSCLC, the prognosis of the patients remains poor. Its five-year survival rate is as low as 15.9% [6], [7]. Thus, there is an increasing awareness to maximize tumor control, prolong overall survival, minimize CT side-effects and improve quality of life (QoL).

In complementary and alternative medicine (CAM), Chinese herbal medicine (CHM) has become increasingly popular for the patients with advanced NSCLC. Recent studies have reported some CHMs in associated with platinum-based CT have definite superiority in relieving the symptoms of lung cancer patients, reducing the severe adverse effects (AEs) of standard cancer therapy, enhancing short-term efficacy and improving patients QoL [8]. There are a variety of herbs being used in different combinations and forms, such as oral administration and intravenous injection, to treat advanced NSCLC combined with platinum-based CT. However, albeit a number of studies being published in Chinese, the evidence on efficacy of CHM as adjuvant therapy to conventional CT is not well demonstrated in the Western world. Thus, the aim of this study is to carry out a comprehensive systematic review about the efficacy of CHM as an adjunctive therapy for advanced NSCLC.

## Methods

Clinical trials were retrieved from 11 databases as well as from conference papers and theses. The studies were reviewed independently by SGL and HYC. The data from included studies were extracted by the first reviewer and verified by the second reviewer. Discrepancies were rectified referring to the original articles. Only the studies which satisfied the criteria were included in the meta-analysis.

### Search Strategy

The terms retrieved in databases were as the following: (non-small-cell lung cancer OR non-small-cell lung carcinoma OR NSCLC OR squamous cell lung carcinoma large cell lung carcinoma OR lung adenocarcinoma) AND (Chinese medicine OR traditional Chinese medicine OR Chinese herbal medicine OR Chinese herbal drug OR traditional herbal medicine OR herbal medicine OR traditional Japanese medicine OR traditional medicine OR ethnomedicine OR folk medicine OR folk remedies OR home remedies OR indigenous medicine OR primitive medicine OR materia medica OR homeopathic remedies OR nosodes OR traditional East Asian medicine OR traditional Far Eastern medicine OR Far East medicine OR Oriental medicine OR Korean medicine OR Tibetan medicine OR herb OR herbaceous agent OR medicinal plant OR medicinal herbs OR medicinal plant product OR plant extract OR plant preparation OR herbal preparation OR botanic OR botany OR Kampo OR Kanpo OR traditional Mongolian medicine OR Mongolian folk medicine OR Mongolian medicine OR phytotherapy OR herb therapy OR herbal therapy OR ethnopharmacology OR ethnobotany OR phytogenic OR alternative medicine OR alternative therapy OR complementary therapy OR complementary medicine OR TCM OR CHM OR Zhong Yi Xue) AND (clinical trial OR randomized controlled trial OR controlled clinical trial OR multicenter study OR phase 1 clinical trial OR phase 2 clinical trial OR phase 3 clinical trial OR phase 4 clinical trial). The terms in Chinese adopted from the above terms were retrieved in Chinese databases.

### Databases

The databases in English language included EMBASE (1974 to September 2012), MEDLINE (1946 to September 2012), AMED (from 1985 to September 2012), EBM Reviews included Cochrane Database of Systematic Reviews (2005 to September 2012), ACP Journal Club (1991 to September 2012), Database of Abstracts of Reviews of Effects (September 2012), Cochrane Central Register of Controlled Trials (September 2012), Cochrane Methodology Register (September 2012), Health Technology Assessment (September 2012), NHS Economic Evaluation Database (September 2012). The databases in Chinese included CNKI (China Knowledge Resource Integrated Database, China academic journals, conference proceedings and theses; 1979 to September 2012).

### Inclusion Criteria

Studies included in the meta-analysis had to meet all of the following criteria: (1) Participants: NSCLC patients had to be diagnosed by pathological sections and were treated by the CT. (2) Type of studies: only clinical randomized controlled trials (RCTs) were eligible. (3) Type of intervention: studies reported CT combined with or without CHM. For studies using other agents as the third arm, only the two arms using CHM and/or CT will be included for analysis. (4) Type of outcome measurements: overall survival rate and tumor response were the main outcome measurements; other outcome measurement included reduction in AEs of CT, improvement in clinical symptoms and blood disorders were also considered.

### Exclusion Criteria

Clinical trials were excluded if they did not meet the above criteria. In addition, studies with the followings were also excluded: (1) CHM were used in both of the intervention group and control group; (2) Non-original research (e.g. review article, letter to the editor); (3) Duplicated publications.

### Outcome Measures

Survival rate, tumor response of CHM on the number of patients with complete response (CR) or partial response (PR), as well as those with progressive disease (PD) based on the WHO scale were examined. The improved or stable performance status of patients were investigated based on the Karnofsky performance score (KPS), in which 100 refers to a normal subject with no complaints, 70 refers to a patient unable to carry on normal activity, 50 refers to a patient who requires considerable assistance, 40 refers to a disabled patient and 30 refers to a hospitalization-recommended patient. The efficacy of CHM on relieving the AEs of CT including nausea and vomiting, hemoglobin (HB), platelet (PLT) were studied by grading the acute and subacute AEs of cancer treatment.

### Quality Assessment

Methodological quality of RCTs was assessed using the five-point Jadad scale [9]. All trials were reviewed by at least two reviewers and any disagreement was resolved by third reviewer consensus. In addition, the risk of bias for the included studies was also assessed.

### Data Analysis

The Review Manager 5.1 software (Nordic Cochrane Centre, Copenhagen, Denmark) was employed for data analysis. The effect data is expressed as relative risk (RR) with 95% confidence interval (CI). If the heterogeneity exists in pooled studies (I^2^>50%), a random model was applied, otherwise the fix model was applied. Statistic significant difference was considered as p<0.05.

## Results

### Characteristics of the Included Studies

**Figure 1:**
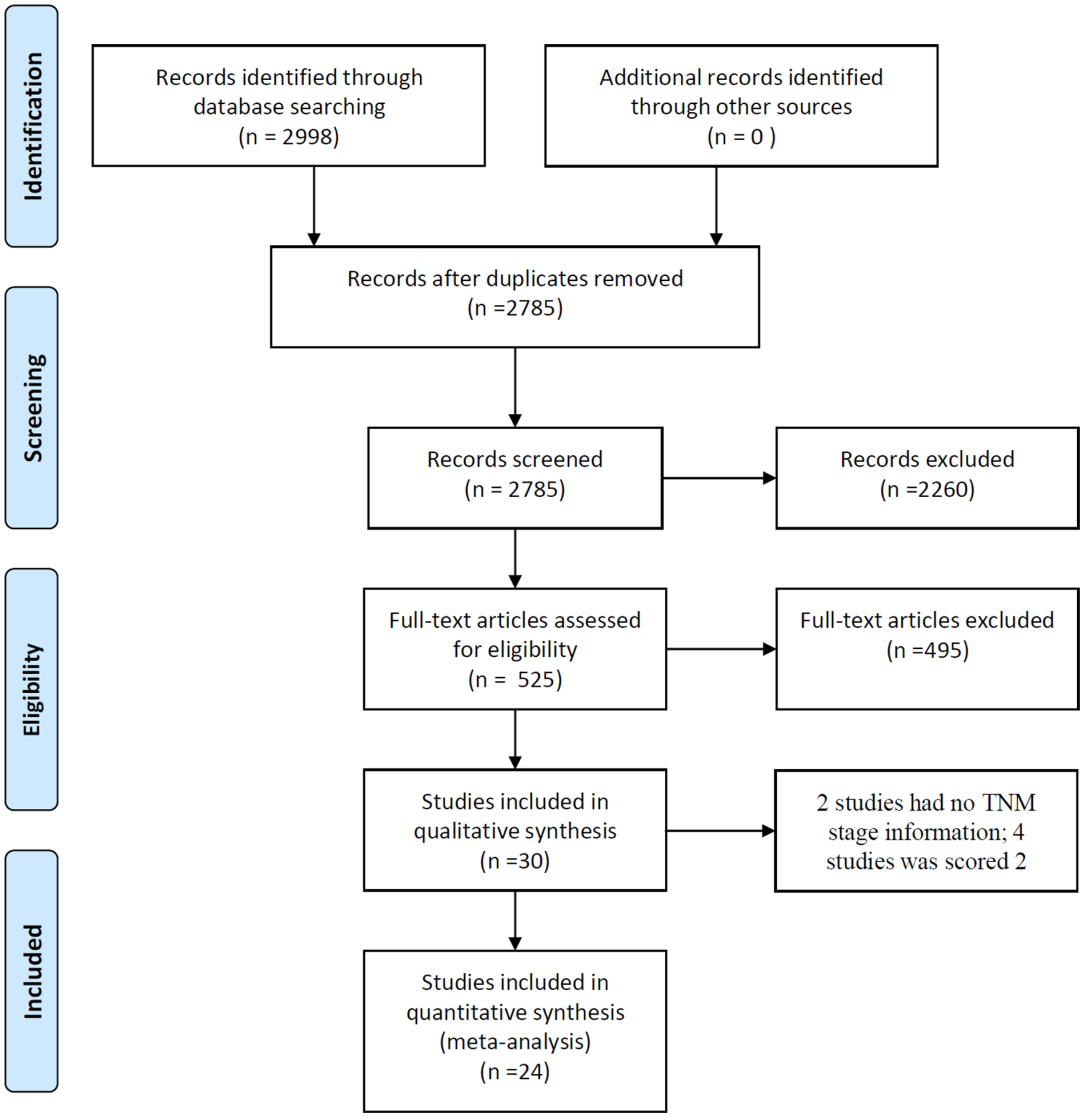
Flow chart of study selection.

In the study, 2,998 articles were retrieved. 30 studies were finally included. Among these studies, six studies were not pooled for analysis as four studies had Jadad score 2 [10]–[13], and other two studies did not report tumor-node-metastasis (TNM) staging information [14], [15]. Therefore, 24 eligible studies were included for meta-analysis. The study selection process details were described in Figure 1. A sum of 2,109 patients was enrolled in these studies, at which 1,064 patients participated in CT combined CHM (CTC) and 1,039 in CT (six patients dropped out and were not reported the group enrolled). A total 78 patients withdraw or dropped out, 36 patients in CTC, 36 in CT and six patients in groups not specified.

All the patients recruited in the 24 studies were at stage III to IV of NSCLC TNM, and all of the studies were graded at least Jadad score 3. The risk of bias of all studies was shown in Table S1. The course of treatment varied from 4 to 16 weeks in the included studies. A list of therapeutic approaches and outcome assessment in each study, and the quality of studies assessed by five-point Jadad scale was listed in Table 1. All studies had claimed the baseline were comparable except one study [32] including age, gender, histopathology or TNM stage as shown in Table S2.

**Table 1:** Characteristics of the included studies.

Study	No. of participants/dropout orwithdrawal	TNM stage	Control groupintervention	CHM formula	Assessment of outcome	Duration (week)	Jadad scale
Chen et al. 2008 [16]	106/6 dropout patients	IIIB–IV	NP	Shengmai injection Gujin grand decoction	Tumor response, survival rate, chemotoxicity	12	3
Chen et al. 2011 [17]	77/0	IIIB–IV	NP/TP	Feiji recipe	Tumor response, survival rate, CD62P	8	3
Deng et al. 2012 [23]	60/drop out: 2 patients in CTC and 3 patients in CT	IIIB–IV	TP/DP	Feitai capsule	Tumor response, CT completion rate, CT delay rate	12	4
Huang et al. 2011 [24]	60/3 withdrawals	IIIB–IV	GP	Yinqi Yangyin decoction	Tumor response, survival rate, chemotoxicity,KPS	8	3
Huang et al. 2012 [25]	60/3 withdrawals	IIIB–IV	GP	Ziyin Qinre Jiedu decoction	Tumor response, chemotoxicity, KPS	11.4	3
Li and Li 2012 [39]	80/1 withdrawal	III–IV	TP	Jianpi Yangxue decoction	Chemotoxicity	3	3
Li et al. 2003 [18]	80/drop out: 7 patients in CTC and 5 patients in CT	III–IV	CAP/EP/CT combined with radiotherapy	Intravenous injection of Xiaoji decoction based on syndrome differentiation	Tumor response, survival rate, chemotoxicity, KPS, CD4, CD8	8	4
Li et al. 2009 [26]	83/0	IIIB–IV	MVP	Haishensu	Tumor response, survival rate, chemotoxicity, KPS, body weight	4	4
Lin 2008 [27]	129/drop out: 5 patients in CTC and4 patients in CT; withdraw: 2 patientsin CTC and 3 patients in CT	IIIB–IV	MVP combined withradiotherapy	Fuzheng Kangai decoction	Tumor response, chemotoxicity,KPS	12	3
Lin and Zheng 2011 [28]	64/drop out: 2 patients in CTC and2 patients in CT	III–IV	NVB/+DDP	Hechan Pian plus CHMs basedon syndrome differentiation	Tumor response, chemotoxicity, body weight	12	3
Lu and Wei 2009 [19]	60/0	III–IV	NP combined withradiotherapy	Shenmai injection	Tumor response, chemotoxicity, survival rate	8	3
Shan et al. 2011 [38]	60/0	III–IV	NP/NC/TP/GP	CHM decoction and intravenous dripping of patent Chinese medicine Yanshu injection	EORTC QLQ-LC43	8	3
Sun 2011 [29]	60/withdraw:1 patient in CT	IIIB–IV	NP	Fuzheng Jiedu decoction	Tumor response, chemotoxicity, KPS, NK cells	9	3
Xu et al. 2007 [30]	120/drop out: 4 patients in CT	III–IV	NP/GP/MVP	Kangliu Zengxiao decoction, Feiyan Ning decoction	Survival rate, KPS, main clinical symptoms, chemotoxicity	12–16	4
Yang 2007 [20]	77/drop out: 4 patients in CTC and3 patients in CT; withdraw: 2 patients in CTC and 2 patients in CT	III–IV	NP	Shengmai injection and Meihua Dianshe pill	Cost-tumor control rate ratio and cost-one year survival rate ratio	8–16	4
Yao et al. 2011 [35]	86/0	III–IV	NP/CE/TP	Yangyin Ruanjian decoction	Quality of life, size of tumor, body weight, survival rate	12	3
Zhang et al. 2008 [22]	120/drop out: 9 patients in CTC and5 patients in CT	III–IV	NP	Artesunate	Tumor response, survival rate, chemotoxicity, mean survival time, time to progression	8	4
Zhang et al. 2012 [21]	135/16 dropouts	IIIB–IV	NP/TP/GP	Feiliuping extract	Therapeutic effect, immune function, KPS, median life span, survival rate of one year	6	4
Zheng et al. 2007 [37]	40/drop out: 2 patients in CTC and 1 patient in CT	III–IV	NP/CE/TP	Feiliuping decoction	NK cytoactivity, CD3, CD4, CD4/CD8, KPS, MDC	8	3
Zheng et al. 2010 [31]	60/0	IIIB–IV	DP	Shenmai injection	Tumor response, chemotoxicity	8	3
Zhou et?al. 2005 [32]	324/30 drop out and withdrawals; CTC and CT were analyzed only. (CTC:103;CT:92; CM:99)	III–IV	NP	Hechan Pian/Shenyi Jiaonang based on syndrome differentiation	Overall response rate, median survival time, cost-effect	12	4
Zhou et al. 2012 [33]	52/2 withdrawals	III–IV	TP/NP/DP/GP	Yiqi Yangyin Huatan decoction based on syndrome differentiation	RECIST evaluation, cost/therapeutic effect ratio	8	3
Zhu and Guo 2011 [34]	182/0	III–IV	TP/NP/GP	Yanshu injection	Tumor response, median survival time, serum tumor markers, chemotoxicity, CD3, CD4, CD4/CD8	16	3
Zhu et al. 2011 [36]	127/3 withdrawals; CTC and CT were analyzed only (CTC: 32; CT:31; other two group: 30, 31 respectively)	III–IV	NP/GP	Kangliu Zengxiao decoction	Clinical symptoms, adverse effect, KPS, NK cytoactivity, CD3, CD4, CD4/CD8	8	4

Abbreviations: CAP = cyclophosphamide+adriamycin+cisplatinum, CHM = Chinese herbal medicine, CT = chemotherapy, CTC = chemotherapy combined with Chinese herb medicine, DDP = cisplatinum, DP = docetaxel+cisplatinum, EP = VP-16+ cisplatinum, GP = gemcitabine+cisplatinum, KPS = Karnofsky performance score, MDC = macrophage-derived chemokine, MVP = mitomycin+vindesine+cisplatinum, NC = vinorelbine+cisplatinum, NP = vinorelbine+cisplatinum, NVB = vinorelbine, RECIST = Response Evaluation Criteria in Solid Tumors, TP = paclitaxel+cisplatinum.

### Survival

**Figure 2:**
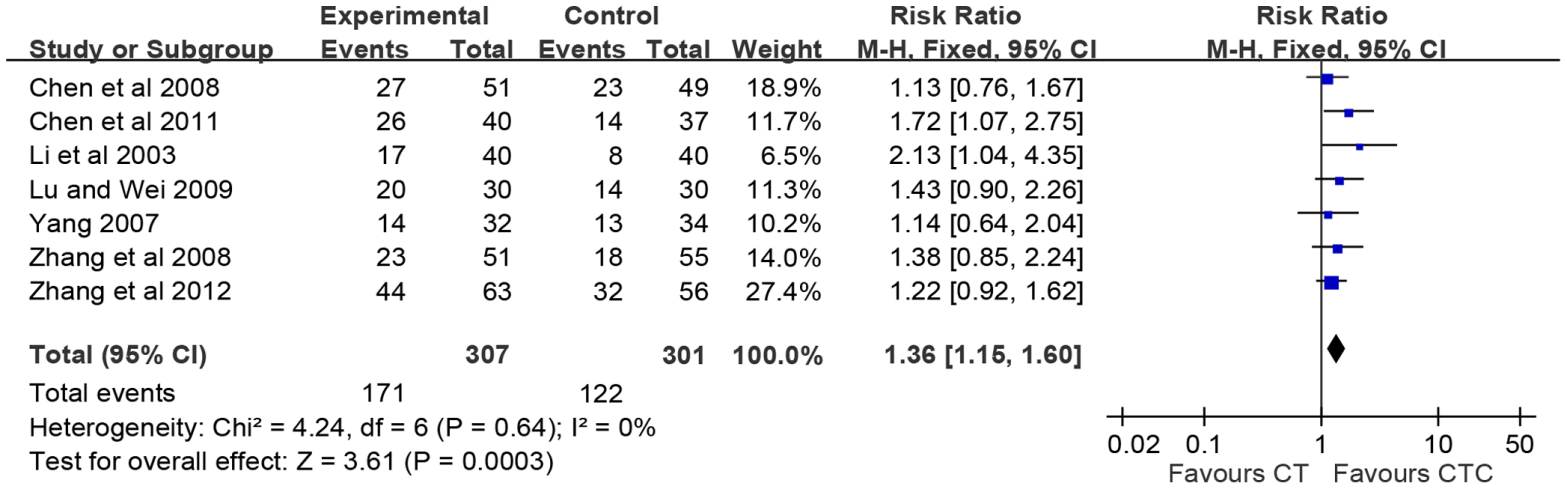
Number of patients with survival >one-year. Overall survivals estimated from meta-analysis of pairwise comparisons in the patients with chemotherapy combined Chinese herbal medicine (CTC, treatment group) versus patients in chemotherapy (CT, control group).

One-year survival was analyzed as shown in Figure 2. One-year survival in pooled studies showed a significant rise in CTC compared to CT alone (RR = 1.36, 95% CI = 1.15–1.60, p = 0.0003, seven studies, 608 patients), with low heterogeneity (I^2^ = 0%) [16]–[22]. A half-year survival also demonstrated a favor of CTC compared to CT alone (RR = 1.18, 95% CI = 1.04–1.33, p = 0.008, two studies, 157 patients) as shown in Figure S1.

### Immediate Tumor Response

**Figure 3:**
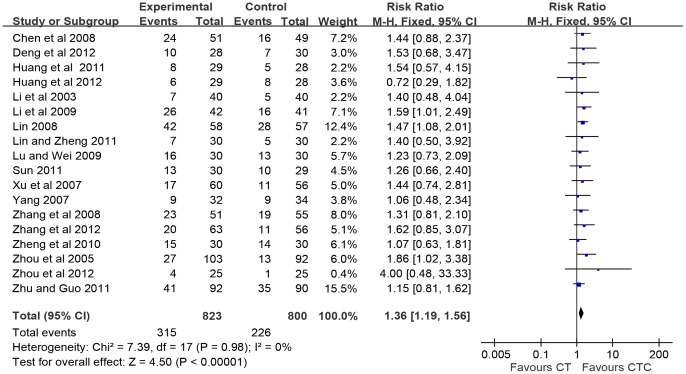
Immediate tumor responses. Immediate tumor responses estimated from meta-analysis of pairwise comparisons in patients with chemotherapy combined Chinese herbal medicine (CTC, treatment group) versus patients in chemotherapy (CT, control group).

As shown in Figure 3, CTC therapy was associated with a significant increase in the number of patients who reported complete or partial response (RR = 1.36, 95% CI = 1.19–1.56, p<1.0E−5, 18 studies, 1,623 patients) [16], [18]–[34]. In addition, the advantage of CTC therapy was found in the number of patients who reported complete, partial and stable response (RR = 1.14, 95% CI = 1.08–1.19, p<1.0E-5, 19 studies, 1,697 patients, figure not showed).

### Performance Status

**Figure 4:**
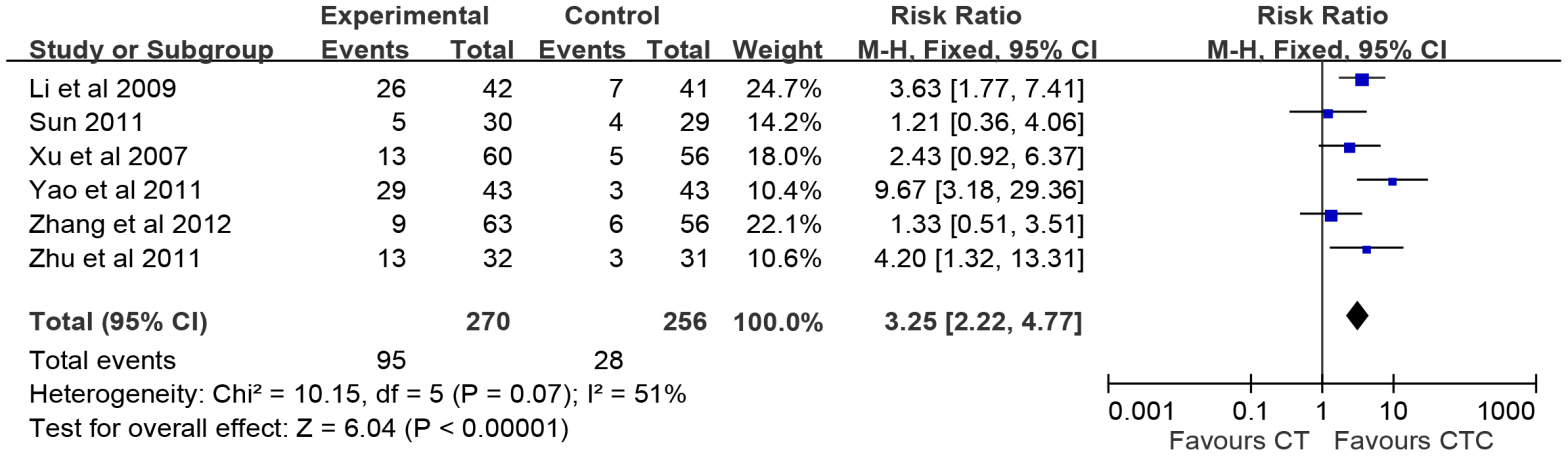
Quality of life. The quality of life changes on Karnofsky performance scale (KPS) were estimated from meta-analysis of pairwise comparisons in patients with Chinese herbal medicine (CTC, treatment group) versus patients in chemotherapy (CT, control group). KPS improvement (the increase of KPS ≥10).

Two types of KPS data were reported in the studies, the improvement of KPS (ten-point cutoff) and the value of KPS in pre- and post-treatment. Six studies of the 24 studies, with evaluation of 526 patients were being analyzed. 35.1% and 10.9% of patients reported improved (the increase of KPS≥10) in CTC (n = 270) and in CT (n = 256), respectively. Significant findings with improvement were shown in the CTC (RR = 2.90, 95% CI = 1.62–5.18, p = 0.0003, six studies, 526 patients) (Figure 4) [21], [26], [29], [30], [35], [36]. There was no significant heterogeneity among these studies (I^2^ = 51%). Dropping one of any studies did not alter the result that favor of CTC. The value of KPS was reported with pre-treatment in seven studies [19], [24]–[26], [30], [34], [37] and post-treatment in four studies [24], [25], [34], [37]. The pooled studies showed that the KPS of pre-treatment had no significant difference in CTC and CT (SMD = −0.04, 95% CI = −0.20-0.12, p = 0.64, I^2^ = 0%). However, the pooled studies indicated the heterogeneity in the four studies of post-treatment (SMD = 1.03, 95% CI = −0.09–2.14, p = 0.07, I^2^ = 95%). Interestingly, these four studies all claimed significant improvement in CTC compared to CT.

### Reduction in CT Toxicity

**Figure 5:**
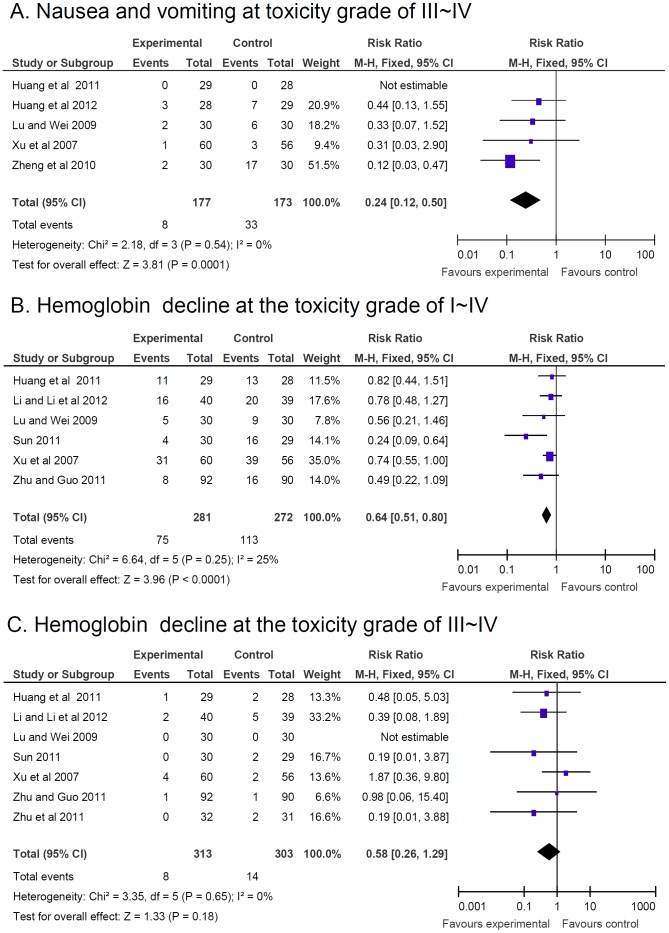
Reduction of adverse effects. Reduction of adverse effects estimated from meta-analysis of pairwise comparisons in patients with Chinese herbal medicine (CHM, treatment group) versus patients without CHM (control group). (**A**) Number of patients with nausea and vomiting at toxicity grade of III-IV. (**B**) Number of patients with hemoglobin decline at the toxicity grade of I-IV with CTC therapy. (**C**) Number of patients with hemoglobin decline at the toxicity grade III–IV with CTC therapy.

Nausea and vomiting are common AEs of CT. A significant reduction of nausea and vomiting at toxicity grade of III–IV in CTC compared to CT therapy was found (RR = 0.24, 95% CI = 0.12–0.50, p = 1.0E−4, five studies, 350 patients) [19], [24], [25], [30], [31] (Figure 5A). However, there was significant heterogeneity in the studies with reduction of nausea and vomiting at toxicity grade of I–IV (data not shown). One study reported a significant reduction of nausea and vomiting in CTC compared to CT at the 10^th^ day of second treatment, based on the questionnaire EORTC QLQ-LC43 (combination of EORTC QLQ-C30 and QLQ-LC13) [38]. The study was not pooled due to the different data types.

The decrease of HB at the toxicity grade of I–IV in patients with CTC therapy was significant reduced (RR = 0.64, 95% CI = 0.51–0.80, p<1.0E−4, six studies, 553 patients, Figure 5B) [19], [24], [29], [30], [34], [39]; yet CTC therapy did not show a significant difference in the decline of HB at the toxicity grade of III–IV (RR = 0.58, 95% CI = 0.26–1.29, p = 0.18, seven studies, 616 patients) compared to CT alone (Figure 5C) [19], [24], [29], [30], [34], [36], [39].

**Figure 6:**
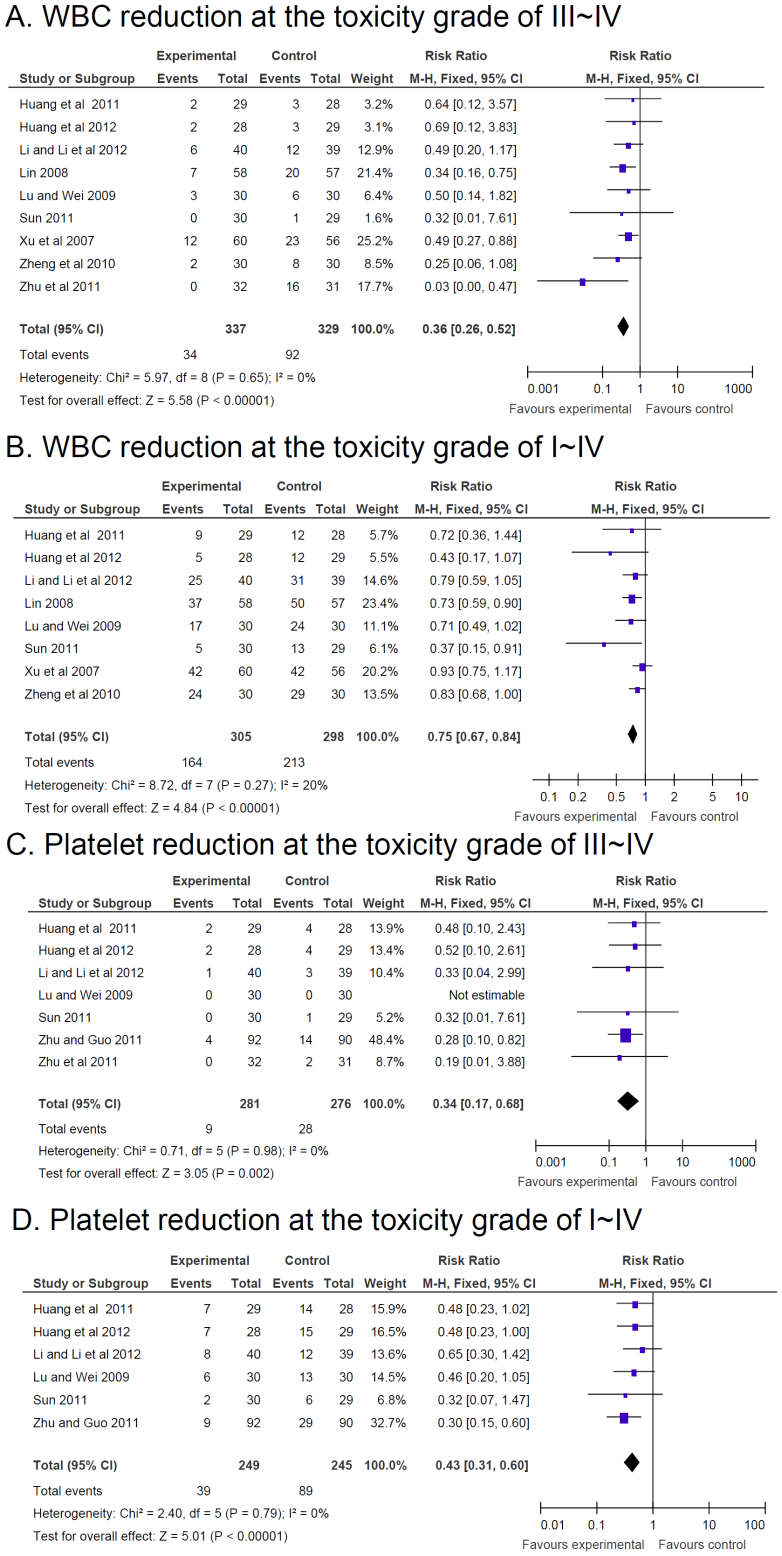
Reduction of adverse effects. Reduction of adverse effects estimated from meta-analysis of pairwise comparisons in patients with Chinese herbal medicine (CHM, treatment group) versus patients without CHM (control group). (**A**) The inhibition of white blood cells (WBCs) at the toxicity grade of III–IV. (**B**) The inhibition of WBCs at the toxicity grade of I–IV. (**C**) The decrease of platelets in numbers at the toxicity grade of III–IV. (**D**) The decrease of platelets in numbers at the toxicity grade of I–IV.

The inhibition of white blood cells (WBCs) at the toxicity grade of III–IV or I–IV in patients with CTC therapy was significant reduced (RR = 0.36, 95% CI = 0.26–0.52, p<1.0E−5, nine studies, 666 patients; RR = 0.75, 95% CI = 0.67–0.84, p<1.0E−5, eight studies, 603 patients, respectively) (Figure 6A and Figure 6B) [19], [24], [25], [27], [29]–[31], [36], [39].

The decrease of PLTs in numbers at the toxicity grade of III–IV was significant prevented in patients with CTC therapy (RR = 0.34, 95% CI = 0.17–0.68, p = 0.002, seven studies, 557 patients) [19], [24], [25], [29], [34], [36], [39]. The decrease of PLTs in numbers at the toxicity grade of III–IV was significant dismissed in patients with CTC therapy (RR = 0.43, 95% CI = 0.31–0.60, p<1E−5, six studies, 494 patients) [19], [24], [25], [29], [34], [39]. In addition, our study showed CTC therapy significantly prevented the decline of PLTs at the toxicity grade of III–IV and I–IV when dropped any one study.

### Herbs Frequently used in NSCLC

19 studies have reported herbs and decoctions. Among them, *Radix Astragalus*, *Radix Adenophorae* and *Radix Ophiopogonis* are the most commonly used herbs for NSCLC (Table 2).

**Table 2:** Herbs frequently used for non-small cell lung cancer.

Chinese herbal medicine	Frequency	
	Count	%
Radix Astragalus	10	52.6
Radix Adenophorae	8	42.1
Radix Ophiopogonis	7	36.8
Radix Glycyrrhizae	5	26.3
Poria	5	26.3
Herba Oldenlandia Diffusa	5	26.3
Radix Asparagi	4	21.1
Semen Persicae	4	21.1
Radix Notoginseng	4	21.1

## Discussion

Recent studies showed that the use of CAM has increasingly gained recognition and usage in cancer patients [40], [41]. CHM is especially popular among the CAM usage as palliative care for cancer patients, but the efficacy of the combined use of CAM and CT on advanced NSCLC cancer remains under explored due to language barrier of many studies reported in Chinese language [42]. Meta-analysis is a powerful statistical analysis of results from individual studies, which increases the precision of a treatment effect and settles controversies studies [43]. In the present study, the pooled data with advanced NSCLC has shown that combined therapy significantly improved the survival rates, immediate tumor response and performance status of advanced NSCLC patients. We also found that, when compared with CT alone, the CTC therapy significantly reduce AEs associated with chemotherapeutic interventions, including nausea and vomiting, decrease in the peripheral blood leukocytes and HB (Figure 5 and Figure 6).

In contrast to most of the previous meta-analyses in this area, our systematic review set the inclusion criteria with Jadad score ≥3 to increase the study quality and included studies with stage III–IV of TNM to minimize the heterogeneity among the studies. It is also encouraging to see that the adjunctive use of CHM with CT may prolong survival in advanced stage NSCLC patients, and CT-related side effects appear to be less frequent and milder in the use of concomitant CAM treatment, which suggest CAM may enhance the compliance to CT and eventually result in improving KPS of patients. The efficacy of CHM as an adjuvant therapy for NSCLC is in line with our previous findings in colorectal cancer, hepatocellular carcinoma and nasopharyngeal carcinoma [44]–[46].

According to Chinese medicine theory, illness is caused by the disharmony of yin and yang, and Chinese medicine aims to restore the balance of yin and yang to alleviate the disease symptoms. CHMs have been commonly used in Asia for thousands of years. Interestingly, it is reported that 61 out of 74 lung cancer patients recruited by 17 Community Clinical Oncology Program (CCOP) affiliates throughout the United States have used CAM while undergoing CT or radiation therapy [47]. However, the potential for integrating CAM into conventional CT treatment remains to be evaluated [48]. *Radix Astragalus, Radix Adenophorae, Radix Ophiopogonis, Radix Glycyrrhizae, Poria and Oldenlandia diffusa* identified in the study have the function of tonifying qi, nourishing yin and removing blood stasis, which are in accordance to the commonest symptoms in NSCLC patients undergoing CT (i.e. blood stasis, vital energy and yin deficiency). It is in line with other findings that *Radix Astragalus, Radix Ophiopogonis and Oldenlandia diffusa* are commonly used for NSCLC [49]. Besides, it has also been reported that there was a significant effectiveness of adding astragalus-based herbal treatment to standard CT regimens [50]. Several experimental researches have revealed that *Adenophora Polysaccharides* and *Oldenlandia diffusa* extract could effectively inhibited the growth of cancer cell lines and induced significant increase of apoptosis. Furthermore, there was a significant inhibition of lung metastases in animal model with no noticeable AEs [51], [52]. *Radix Adenophorae* and *Radix Ophiopogonis* were also shown to have anti-inflammation and immunomodulating effects [53], [54]. Our previous studies have demonstrated that *Radix Astragalus*, the dried root of *Astragalus membranaceus*, has anti-tumor, immunomodulating and immunorestorative effects *in vivo* and *in vitro* [55], [56]. It is in accordance with other findings that *Radix Astragulus* has immunologic benefits by stimulating macrophage, natural killer cell activity but inhibiting T-helper cell type 2 cytokines [57]. In addition, the combination of *Radix Astragulus* and *Radix Angelicae* increased WBC, HB and PLT in cyclophosphamide-induced anemic rat [58]. This study also indicated that these two herbs are associated with enhancing erythropoietin expression [58]. Erythropoietin is also able to prevent against cisplatin cytotoxicity in cells via several mechanisms [59], [60]. These findings may give some insights on the mechanism of how CHM improved the hematological parameters in this systematic review. Furthermore, *Radix Astragalus* also reduced toxicity induced by cyclophosphamide [61]. Though the molecular mechanism is not fully understood, the immunostimulating effects and the reduction of chemotherapy-induced toxicity may be the two major advantages for CHM as adjuvant therapy in NSCLC treatment.

Survival rate, immediate tumor response and chemo-toxicity are three major outcomes in the studies. However, not all the studies simultaneously reported the three outcomes. For example, Zhang et al. [21] reported all three outcomes while Li and Li [39] reported the reduction of chemo-toxicity only. Nevertheless, we analyzed all available data in these reports without any subjective selection. KPS is a scale for the evaluation of cancer patients. In this study, we analyzed continuous data (average of KPS scores) and discontinuous data (patient number with an increase of KPS≥10). There is significant heterogeneity when we pooled studies with continuous data, although the baseline has no heterogeneity in the meta-analysis. The variation of KPS value in the studies may be due to the differences in treatment duration, treatment methods and the herb prescriptions in different studies. Therefore, the change of KPS in patients is more accurate to indicate the efficacy of treatments in pooled studies. The interval of ten in KPS normally shows a significant change of performance status as described by previous publications [26], [57], [62], [63]. Hence, we adopted a ten-point increase as the cutoff for improved performance status. The findings showed a significant improvement in KPS with CTC treatment.

In conclusion, the evidence from the meta-analysis of the included studies shows that CHM as adjuvant therapy has advantages in NSCLC patients. However, due to the complex nature of CHM interventions, particular attention should be paid to apply appropriate and rigorous research methodologies to investigate CHM as a holistic system [64]. Therefore a large scale RCT integrated the Chinese Medicine methodology of pattern diagnosis and treatment is warranted for further study.

## Supporting Information

Figure S1 **Number of patients with survival >half-year.** Overall survivals estimated from meta-analysis of pairwise comparisons in the patients with chemotherapy combined Chinese herbal medicine (CTC, treatment group) versus patients in chemotherapy (CT, control group).
(TIF)

Table S1 The risk of bias of the included studies.
(DOC)

Table S2 Age, gender and baseline of studies.
(DOC)

## References

[1] Siegel R, Naishadham D, Jemal A (2012) Cancer statistics, 2012. CA Cancer J Clin 62: 10–29. 22237781 10.3322/caac.20138

[2] Pfister DG, Johnson DH, Azzoli CG, Sause W, Smith TJ, et al (2004) American Society of Clinical Oncology treatment of unresectable non-small-cell lung cancer guideline: update 2003. J Clin Oncol 22: 330–353. 14691125 10.1200/JCO.2004.09.053

[3] Azzoli CG, Temin S, Aliff T, Baker S Jr, Brahmer J, et al (2011) 2011 Focused update of 2009 American Society of Clinical Oncology Clinical practice guideline update on chemotherapy for stage IV non-small-cell lung cancer. J Clin Oncol 29: 3825–3831. 21900105 10.1200/JCO.2010.34.2774 PMC3675703

[4] Arriagada R, Dunant A, Pignon JP, Bergman B, Chabowski M, et al (2010) Long-term results of the international adjuvant lung cancer trial evaluating adjuvant cisplatin-based chemotherapy in resected lung cancer. J Clin Oncol 28: 35–42. 19933916 10.1200/JCO.2009.23.2272

[5] Kato H, Tsuboi M, Kato Y, Ikeda N, Okunaka T, et al (2005) Postoperative adjuvant therapy for completely resected early-stage non-small cell lung cancer. Int J Clin Oncol 10: 157–164. 15990962 10.1007/s10147-005-0493-x

[6] Gkiozos I, Charpidou A, Syrigos K (2007) Developments in the treatment of non-small cell lung cancer. Anticancer Res 27: 2823–2827. 17695454

[7] Baggstrom MQ, Stinchcombe TE, Fried DB, Poole C, Hensing TA, et al (2007) Third-generation chemotherapy agents in the treatment of advanced non-small cell lung cancer: a meta-analysis. J Thorac Oncol 2: 845–853. 17805063 10.1097/JTO.0b013e31814617a2

[8] Chen YZ, Li ZD, Gao F, Zhang Y, Sun H, et al (2009) Effects of combined Chinese drugs and chemotherapy in treating advanced non-small cell lung cancer. Chin J Integr Med 15: 415–419. 20082245 10.1007/s11655-009-0415-2

[9] Jadad AR, Moore RA, Carroll D, Jenkinson C, Reynolds DJ, et al (1996) Assessing the quality of reports of randomized clinical trials: is blinding necessary? Control Clin Trials 17: 1–12. 8721797 10.1016/0197-2456(95)00134-4

[10] Huang C, Liao TH (2008) Effect of combination of Chenxialiujun decoction and chemotherapy on non-small lung cell cancer. Hebei J Tradit Chin Med 30: 1287–1288.

[11] Liang QL, Zhang Y, Xie JR, Li SJ, Luo HQ, et al (2007) Clillical study on effcacy of compound Dashen droplet pill with chemotherapy in treating advanced non-small cell lung cancer. Shanghai J Tradit Chin Med 41: 22–24.

[12] Qi YF, Li XR, Li HJ (2011) Clinical study on lung cancer treated by Dahuang Zhechong Wan. Chin J Inf Tradit Chin Med 18: 15–17.

[13] Yan GY, Xu ZY, Deng HB, Wan ZY, Zhang L, et al (2011) Effect s of chemotherapy combined with Chinese herbal medicine Kangliu Zengxiao decoction on tumor markers of patient s with advanced non-small cell lung cancer: a randomized, controlled trial. J Chin Integr Med 9: 525–530. 10.3736/jcim20110509 21565138

[14] Yang PY, Jia YJ, Chen J, Li XJ, Sun YY, et al (2011) A clinical study of Xiao-yan decoction combined with chemotherapy on the immune function of the NSCLC patients with “Qi deficiency and blood statsis”. J New Chin Med 43: 64–65.

[15] Wang YC, Zhang RH (2009) Clinical effect of non-small cell lung cancer treated by combined chemotherapy with Zhongliuan capsule. Chin Tradit Patent Med 31: 669–673.

[16] Chen Y, Di L, Zhang S, Chen M, Sun H, et al (2008) The randomized, multicenter, controlled clinic trail for treating advanced non-small cell lung cancer with combined traditional chinese medicine and vinorelbine (Navelbine, NVB) plus cisplatin (DDP) chemotherapy. Zhongguo Fei Ai Za Zhi 11: 441–444. 20731951 10.3779/j.issn.1009-3419.2008.03.003

[17] Chen XZ, Huang ZQ, Yang YL, Qian HQ, Liang GW, et al (2011) The intervention effect of Feiji recipe on CD62P of non-small cell lung cancer patients. Chin Med Mod Distance Ed China 9: 93–95.

[18] Li LN, Liu WS, Xu K, Wu WY, Liu YL, et al (2003) The prognosis factors of comprehensive treatment with traditional Chinese medicine to non-small cell lung cancer. Shanxi Cancer Med 11: 44–47.

[19] Lu XA, Wei YR (2009) Therapeutic effect of combination of Shenmai injection and radio-chemotherapy on advanced non-small cell lung cancer. Hebei J Tradit Chin Med 31: 597–599.

[20] Yang HL (2007) Cost-effect analysis of integrated Chinese and Western medical treatment for advanced non-small cell lung cancer. J Tradit Chin Med 48: 232–234.

[21] Zhang PT, Lin HS, Yu MW, Yang ZY, Dong HT, et al (2012) Application of traditional Chinese medicine and Western medical therapeutic evaluation methodologies for the combination treatment with Feiliuping extract and chemotherapy in advanced non-small cell lung cancer. J Tradit Chin Med 53: 403–406.

[22] Zhang Z, Yu SQ, Miao L, Huang XY, Zhang XP, et al (2008) Artesunate combined with vinorelbline plus cisplatin in treatment of advanced non-small cell lung cancer: a randomized controlled trial. Zhong Xi Yi Jie He Xue Bao 6: 134–138. 18241646 10.3736/jcim20080206

[23] Deng S, Ouyang X, Yu Z, Dai X, Chen X, et al (2012) Influence of Chinese herbal medicine Feitai capsule on completion or delay of chemotherapy in patients with stage IIIB/IV non-small cell lung cancer: a randomized controlled trial. Zhong Xi Yi Jie He Xue Bao 10: 635–640. 22704411 10.3736/jcim20120606

[24] Huang YL, Hou AJ, Hu Y, Zhou L, Gao HF (2011) The clinical effect of Yi Qi Yang Yin Chinese medicine combined with GP chemotherapy to the advanced non-small cell lung cancer patients. J New Chin Med 43: 47–49.

[25] Huang YL, Hou AJ, Zhou L, Hu Y, Shen XY (2012) Clinical study on non-small cell lung cancer treated by Zi Yin Qin Re herbs combined with chemotherapy. Liaoning Tradit Chin Med J 2: 53.

[26] Li GY, Yu XM, Zhang HW, Zhang B, Wang CB, et al (2009) Haishengsu as an adjunct therapy to conventional chemotherapy in patients with non-small cell lung cancer: a pilot randomized and placebo-controlled clinical trial. Complement Ther Med 17: 51–55. 19114229 10.1016/j.ctim.2008.10.002

[27] Lin J (2008) Clinical study on the combined Chinese and Western medicine in treating 58 cases of non-small cell lung cancer. Henan J Tradit Chin Med 28: 29–31.

[28] Lin LZ, Zheng XT (2011) Clinical observation of the therapeutic effect of traditional Chinese medicine in the treatment of elderly non-small cell lung cancer. Tradit Chin Drug Res Clin Pharmacol 22: 120–122.

[29] Sun QS (2011) Clinical observation of the therapeutic effect of chemotherapy plus traditional Chinese medicine in the treatment of advanced non-small cell lung cancer. Hainan Med J 22: 31–33.

[30] Xu ZY, Jin CJ, Shen DY, Li M, Zhou WD, et al (2007) Clinical study on treatment of advanced non-small cell lung cancer with Chinese herbal medicine in different stages combined with chemotherapy. Zhongguo Zhong Xi Yi Jie He Za Zhi 27: 874–878. 17990450

[31] Zheng QH, Liao XF, Wang Y (2010) Thirty cases of docetaxel plus cisplatin with Shenmai injection in treatment of advanced non-small cell lung cancer. Jiangxi J Tradit Chin Med 57–58.

[32] Zhou DH, Lin LZ, Zhou YQ, Luo RC, LIu KF, et al (2005) Cost-effectiveness analysis of three therapeutic regimens for advanced non-small cell lung cancer. Chin J Clin Oncol 32: 1081–1084.

[33] Zhou L, Liu JX, Li HG, Zhao LH, Liu LS, et al (2012) Comparative study on treatment of advance non-small cell lung cancer by two methods. Shanghai J Tradit Chin Med 5: 8.

[34] Zhu LM, Guo JF (2011) Clinical study of Yanshu injection on treating advanced non-small cell lung cancer. Chin J Clin Oncol Rehabil 18: 380–384.

[35] Yao QB, Wang LX, Ni W, Shen YL, Zhu Y, et al (2011) Clinical observation of “Yang Yin Ruan Jian” on non-small cell lung cancer. J Zhejiang Chin Med Uni 35: 501–503.

[36] Zhu LM, Zhang H, Zhou H, Pan CF, Shen KP, et al (2011) Time-effect relationship of Kangliu Zengxiao decoction in reducing chemotherapy associated adverse reactions in patients with advanced non-small cell lung cancer. J Shanghai Jiaotong Uni (Med Sci) 31: 797–801.

[37] Zheng HG, Pu BK, Lin HS, Xiong L, Hua BJ, et al (2007) The efficacy of Feiliuping decoction on dendritic cell subtypes and immune function in patients with non-small cell lung carcinoma. Beijing J Tradit Chin Med 26: 214–217.

[38] Shan M, Han B, You J (2011) Assessment of therapeutic efficacy on treating advanced non-small cell lung cancer in the aged by Chinese medicine adopting the international questionnaire of quality of life. Zhongguo Zhong Xi Yi Jie He Za Zhi 31: 873–879. 21866652

[39] Li Y, Li C (2012) Clinical study of treatment of cancer chemotherapy by Jianpi Yangxue Tang. Guangming J Chin Med 27: 108–109.

[40] Hyodo I, Amano N, Eguchi K, Narabayashi M, Imanishi J, et al (2005) Nationwide survey on complementary and alternative medicine in cancer patients in Japan. J Clin Oncol 23: 2645–2654. 15728227 10.1200/JCO.2005.04.126

[41] Chang KH, Brodie R, Choong MA, Sweeney KJ, Kerin MJ (2011) Complementary and alternative medicine use in oncology: a questionnaire survey of patients and health care professionals. BMC Cancer 11: 196. 21609461 10.1186/1471-2407-11-196 PMC3123324

[42] Li JH (1996) A study on treatment of lung cancer by combined therapy of traditional Chinese medicine and chemotherapy. Zhongguo Zhong Xi Yi Jie He Za Zhi 16: 136–138. 9208532

[43] Bent S (2008) Herbal medicine in the United States: review of efficacy, safety, and regulation: grand rounds at University of California, San Francisco Medical Center. J Gen Intern Med 23: 854–859. 18415652 10.1007/s11606-008-0632-y PMC2517879

[44] Cho WC, Chen HY (2009) Transcatheter arterial chemoembolization combined with or without Chinese herbal therapy for hepatocellular carcinoma: meta-analysis. Expert Opin Investig Drugs 18: 617–635. 10.1517/13543780902855308 19388879

[45] Cho WC, Chen HY (2009) Clinical efficacy of traditional Chinese medicine as a concomitant therapy for nasopharyngeal carcinoma: a systematic review and meta-analysis. Cancer Invest 27: 334–344. 19212827 10.1080/07357900802392683

[46] Zhong LL, Chen HY, Cho WC, Meng X, Tong Y (2012) The efficacy of Chinese herbal medicine as an adjunctive therapy for colorectal cancer: a systematic review and meta-analysis. Complementary therapies in medicine. Complement Ther Med 20: 240–252. 22579437 10.1016/j.ctim.2012.02.004

[47] Yates JS, Mustian KM, Morrow GR, Gillies LJ, Padmanaban D, et al (2005) Prevalence of complementary and alternative medicine use in cancer patients during treatment. Supportive Care Cancer 13: 806–811. 10.1007/s00520-004-0770-7 15711946

[48] Brake MK, Bartlett C, Hart RD, Trites JR, Taylor SM (2011) Complementary and alternative medicine use in the thyroid patients of a head and neck practice. Otolaryngol Head Neck Surg 145: 208–212. 21521893 10.1177/0194599811407564

[49] Chen S, Flower A, Ritchie A, Liu J, Molassiotis A, et al (2010) Oral Chinese herbal medicine (CHM) as an adjuvant treatment during chemotherapy for non-small cell lung cancer: a systematic review. Lung Cancer 68: 137–145. 20015572 10.1016/j.lungcan.2009.11.008

[50] Dugoua JJ, Wu P, Seely D, Eyawo O, Mills E (2010) Astragalus-containing Chinese herbal combinations for advanced non-small-cell lung cancer: a meta-analysis of 65 clinical trials enrolling 4751 patients. Lung Cancer 1: 85–100. 28210109 10.2147/lctt.s7780 PMC5312465

[51] Li Y, Li C-H, Tang F-T, Li X-F (2004) Pharmacological action of adenophora polysaccharides. Chin J Integr Med 10: 78–80.

[52] Gupta S, Zhang D, Yi J, Shao J (2004) Anticancer activities of Oldenlandia diffusa. J Herb Pharmacother 4: 21–33. 15273074

[53] Wu X, Dai H, Huang L, Gao X, Tsim KW, et al (2006) A fructan, from Radix Ophiopogonis, stimulates the proliferation of cultured lymphocytes: structural and functional analyses. J Nat Products 69: 1257–1260. 10.1021/np060033d 16989515

[54] Roh SS, Kim SH, Lee YC, Seo YB (2008) Effects of Radix Adenophorae and cyclosporine A on an OVA-induced murine model of asthma by suppressing to T cells activity, eosinophilia, and bronchial hyperresponsiveness. Mediators Inflamm 2008: 781425. 18382613 10.1155/2008/781425 PMC2276601

[55] Cho WC, Leung KN (2007) In vitro and in vivo immunomodulating and immune restorative effects of Astragalus membranaceus. J Ethnopharmacol 113: 132–141. 17611061 10.1016/j.jep.2007.05.020

[56] Cho WC, Leung KN (2007) In vitro and in vivo anti-tumor effects of Astragalus membranaceus. Cancer Lett 252: 43–54. 17223259 10.1016/j.canlet.2006.12.001

[57] McCulloch M, See C, Shu XJ, Broffman M, Kramer A, et al (2006) Astragalus-based Chinese herbs and platinum-based chemotherapy for advanced non-small-cell lung cancer: meta-analysis of randomized trials. J Clin Oncol 24: 419–430. 16421421 10.1200/JCO.2005.03.6392

[58] Chang MS, Kim do R, Ko EB, Choi BJ, Park SY, et al (2009) Treatment with Astragali radix and Angelicae radix enhances erythropoietin gene expression in the cyclophosphamide-induced anemic rat. J Med Food 12: 637–642. 19627214 10.1089/jmf.2007.0727

[59] Rjiba-Touati K, Ayed-Boussema I, Belarbia A, Azzebi A, Achour A, et al (2013) Protective effect of recombinant human erythropoeitin against cisplatin cytotoxicity and genotoxicity in cultured Vero cells. Exp Toxicol Pathol 65: 181–187. 21924599 10.1016/j.etp.2011.08.004

[60] Li J, Vesey DA, Johnson DW, Gobe G (2007) Erythropoietin reduces cisplatin-induced apoptosis in renal carcinoma cells via a PKC dependent pathway. Cancer Biol Ther 6: 1944–1950. 18075299 10.4161/cbt.6.12.4975

[61] Kim W, Kim SH, Park SK, Chang MS (2012) Astragalus membranaceus ameliorates reproductive toxicity induced by cyclophosphamide in male mice. Phytother Res 26: 1418–1421. 22674751 10.1002/ptr.4756

[62] Yates JW, Chalmer B, McKegney FP (1980) Evaluation of patients with advanced cancer using the Karnofsky performance status. Cancer 45: 2220–2224. 7370963 10.1002/1097-0142(19800415)45:8<2220::aid-cncr2820450835>3.0.co;2-q

[63] Glare P (2005) Clinical predictors of survival in advanced cancer. J Support Oncol 3: 331–339. 16218255

[64] Flower A, Witt C, Liu JP, Ulrich-Merzenich G, Yu H, et al (2012) Guidelines for randomised controlled trials investigating Chinese herbal medicine. J Ethnopharmacol 140: 550–554. 22210103 10.1016/j.jep.2011.12.017

