# Supplementary figures and images for: The Efficacy of Chinese Herbal Medicine as an Adjunctive Therapy for Advanced Non-small Cell Lung Cancer: A Systematic Review and Meta-analysis
Source: PLoS One. 2013 Feb 28;8(2):e57604. doi: 10.1371/journal.pone.0057604 (PMC3585199; doi:10.1371/journal.pone.0057604)

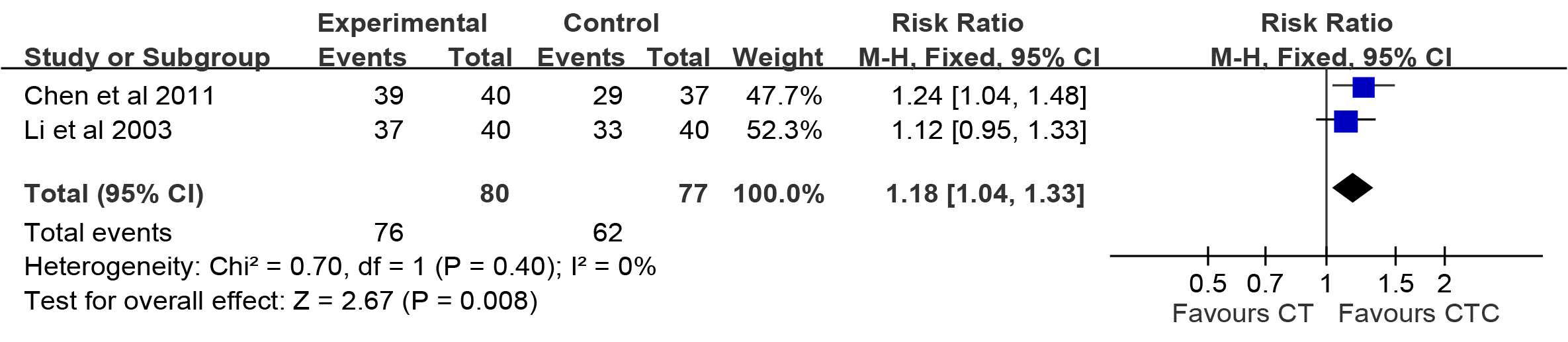

Supplement: Figure S1 — Number of patients with survival >half-year. Overall survivals estimated from meta-analysis of pairwise comparisons in the patients with chemotherapy combined Chinese herbal medicine (CTC, treatment group) versus patients in chemotherapy (CT, control group). (TIF) [file pone.0057604.s001.tif]
