# Supplementary material for: The Efficacy of Chinese Herbal Medicine as an Adjunctive Therapy for Advanced Non-small Cell Lung Cancer: A Systematic Review and Meta-analysis
Source: PLoS One. 2013 Feb 28;8(2):e57604. doi: 10.1371/journal.pone.0057604 (PMC3585199; doi:10.1371/journal.pone.0057604)
Supplement: Table S1 — The risk of bias of the included studies. (DOC) [file pone.0057604.s002.doc]

Table S1. The risk of bias of the included studies

| **Studies** | **Selection bias** | | **Performance bias** | **Detection bias** | **Attrition bias** | **Reporting bias** | **Other bias** |
| --- | --- | --- | --- | --- | --- | --- | --- |
|  | **Random sequence generation** | **Allocation concealment** | **Blinding of participants and personnel** | **Blinding of outcome assessment** | **Incomplete outcome data** | **Selective reporting** | **Other sources of bias** |
| Chen et al. 2008 [16] | L | L | U | U | L | U | L |
| Chen et al. 2011 [17] | L | U | U | U | L | U | L |
| Deng et al. 2012 [23] | L | U | U | U | L | U | L |
| Huang et al. 2011 [24] | L | L | U (single-blind) | U | L | U | L |
| Huang et al. 2012 [25] | U | U | U | U | L | U | L |
| Li and Li 2012 [39] | L | L | U | U | L | U | L |
| Li et al. 2003 [18] | L | U | U (single-blind) | U | L | U | L |
| Li et al. 2009 [26] | L | L | L (double-blind) | L | L | U | L |
| Lin 2008 [27] | L | U | U | U | L | U | L |
| Lin and Zheng 2011 [28] | L | U | U | U | L | U | L |
| Lu and Wei 2009 [19] | L | U | U | U | L | U | L |
| Shan et al. 2011 [38] | L | U | U | U | L | U | L |
| Sun 2011 [29] | L | U | U (single-blind) | U | L | U | L |
| Xu et al. 2007 [30] | L | L | U (single-blind) | U | L | U | L |
| Yang 2007 [20] | L | U | U | U | L | U | L |
| Yao et al. 2011 [35] | L | U | U | U | L | U | L |
| Zhang et al. 2008 [22] | L | U | U | U | L | U | L |
| Zhang et al. 2012 [46] | L | L | U | U | L | U | L |
| Zheng et al. 2007 [37] | L | U | U | U | L | U | L |
| Zheng et al. 2010 [31] | L | L | U | U | L | U | L |
| Zhou et al. 2005 [32] | L | U | U | U | L | U | L |
| Zhou et al. 2012 [33] | L | U | U | U | L | U | L |
| Zhu and Guo 2011 [34] | L | U | U | U | L | U | L |
| Zhu et al. 2011 [36] | L | L | U (single-blind) | U | L | U | L |

L, low risk, U, unclear risk
