# Supplementary material for: The Efficacy of Chinese Herbal Medicine as an Adjunctive Therapy for Advanced Non-small Cell Lung Cancer: A Systematic Review and Meta-analysis
Source: PLoS One. 2013 Feb 28;8(2):e57604. doi: 10.1371/journal.pone.0057604 (PMC3585199; doi:10.1371/journal.pone.0057604)
Supplement: Table S2 — Age, gender and baseline of studies. (DOC) [file pone.0057604.s003.doc]

**Table S2. Age, gender and baseline of studies**

| **Studies** |  | **CTC** | | | |  | **CT** | | | | |  | **Baseline (no significant difference in CTC and CT)** |
| --- | --- | --- | --- | --- | --- | --- | --- | --- | --- | --- | --- | --- | --- |
|  |  | **Age (years)** | **Gender** | | |  | **Age (years)** |  | **Gender** | | |  |  |
|  |  |  | **M** | | **F** |  |  |  | **M** | **F** | |  |  |
| Chen et al. 2008 [16] |  | 36~81, median 62.8 | 29 | | 22 |  | 29~75, median 63.5 |  | 33 | 16 | |  | age, gender, histopathology, ECOG PS, TNM stage |
| Chen et al. 2011 [17] |  | 61.5 ± 8.1 | 29 | | 11 |  | 64.5 ± 8.8 |  | 25 | 12 | |  | age, gender |
| Deng et al. 2012 [23] |  | 36~74, median 59.5 | 23 | | 5 |  | 29~72, median 55.7 |  | 24 | 3 | |  | age, gender, TNM stage, KPS |
| Huang et al. 2011 [24] |  | >40 | 23 | | 7 |  | >40 |  | 25 | 5 | |  | age, gender, TNM stage, ECOG PS, KPS |
| Huang et al. 2012 [25] |  | >40 | 21 | | 9 |  | >40 |  | 24 | 6 | |  | age, gender, TNM stage, PS, KPS |
| Li and Li 2012 [39] |  | 38~72, median 58.7 | 27 | | 13 |  | 36~73, median 56.9 |  | 28 | 11 | |  | age, gender, histopathology, TNM stage |
| Li et al. 2003 [18] |  | 18~75 | 25 | | 15 |  | 18~75 |  | 22 | 18 | |  | age, gender, histopathology, TNM stage, KPS |
| Li et al. 2009 [26] |  | 59.3 ± 5.9 | 33 | | 9 |  | 59.2 ± 5.8 |  | 32 | 9 | |  | age, gender, histopathology, TNM stage, body weight, KPS |
| Lin 2008 [27] |  | 44~73, median 58 | 40 | | 18 |  | 45~71, median 59 |  | 41 | 16 | |  | age, gender, histopathology, TNM stage |
| Lin and Zheng 2011 [28] |  | 70.88 ± 3.41 | 22 | | 8 |  | 70.21 ± 2.96 |  | 20 | 10 | |  | age, gender, histopathology, TNM stage |
| Lu and Wei 2009 [19] |  | 51.64 ± 9.18 | 22 | | 8 |  | 56.58 ± 9.97 |  | 24 | 6 | |  | age, gender, histopathology, TNM stage, KPS |
| Shan et al. 2011 [38] |  | 70.9 ± 4.5 | 19 | | 11 |  | 70.33 ± 3.62 |  | 22 | 8 | |  | age, gender, histopathology, TNM stage |
| Sun 2011 [29] |  | 43~67, median 55 | 19 | | 11 |  | 44~68, median 54 |  | 21 | 9 | |  | age, gender, histopathology |
| Xu et al. 2007 [30] |  | 64.17 ± 10.71 | 42 | | 18 |  | 62.38 ± 9.22 |  | 37 | 19 | |  | age, gender, TNM stage, KPS |
| Yang 2007 [20] |  | 62.69 ± 9.12 | 22 | | 10 |  | 58.91 ± 7.77 |  | 24 | 10 | |  | age, gender, histopathology |
| Yao et al. 2011 [35] |  | 69.25 ± 12.16 | 25 | | 18 |  | 71.31 ± 10.42 |  | 27 | 16 | |  | age, gender, body weight, histopathology |
| Zhang et al. 2008 [22] |  | 59.3 ± 10.6 | 35 | | 16 |  | 61.6 ± 11.2 |  | 37 | 18 | |  | age, gender, histopathology, TNM stage, ECOG PS |
| Zhang et al. 2012 [46] |  | 62.54 ± 9.39 | 41 | | 22 |  | 62.46 ± 9.75 |  | 37 | 19 | |  | age, gender |
| Zheng et al. 2007 [37] |  | 67.55 ± 8.49 | 10 | | 8 |  | 64.82 ± 9.17 |  | 11 | 8 | |  | age, gender, histopathology, TNM stage, KPS |
| Zheng et al. 2010 [31] |  | 42~80, median 60.50 | 23 | | 7 |  | 35~83, median 63.65 |  | 22 | 8 | |  | age, gender, histopathology, TNM stage, KPS |
| Zhou et al. 2005 [32] |  | 28~80, median 60 | 103 | | |  | 28~80, median 60 |  | 92 | | |  | Unclear |
| Zhou et al. 2012 [33] |  | 61.72 ± 8.19 | 14 | 11 | |  | 62.52 ± 8.57 |  | 20 | | 7 |  | age, gender, histopathology, TNM stage |
| Zhu and Guo 2011 [34] |  | 64.7 ± 12.5 | 53 | 39 | |  | 63.9 ± 11.9 |  | 51 | | 39 |  | age, gender, histopathology, TNM stage, ECOG PS |
| Zhu et al. 2011 [36] |  | 62.1 ± 8.47 | 20 | 12 | |  | 62.2 ± 8.51 |  | 20 | | 11 |  | age, gender, histopathology, TNM stage, KPS |

Abbreviations: CTC = chemotherapy combined with Chinese herb medicine, CT = chemotherapy, ECOG = Eastern Cooperative Oncology Group, KPS = Karnofsky performance score, PS = performance status, TNM = tumor-node-metastasis.
